# Vegetarian Diet during Pregnancy Is Not Associated with Poorer Cognitive Performance in Children at Age 6–7 Years

**DOI:** 10.3390/nu11123029

**Published:** 2019-12-11

**Authors:** Sarah R. Crozier, Keith M. Godfrey, Philip C. Calder, Sian M. Robinson, Hazel M. Inskip, Janis Baird, Catharine R. Gale, Cyrus Cooper, Charlene M. Sibbons, Helena L. Fisk, Graham C. Burdge

**Affiliations:** 1 MRC Lifecourse Epidemiology Unit, Southampton General Hospital, University of Southampton, Southampton SO16 6YD, UK; 2 NIHR Southampton Biomedical Research Centre, University Hospital Southampton NHS Foundation Trust and University of Southampton, Southampton SO16 6YD, UK; 3 School of Human Development and Health, Faculty of Medicine, University of Southampton, Southampton SO16 6YD, UK; 4 AGE Research Group, Newcastle University, Newcastle upon Tyne NE4 5PL, UK; 5 NIHR Newcastle Biomedical Research Centre, Newcastle upon Tyne Hospitals NHS Foundation Trust and Newcastle University, Newcastle upon Tyne NE4 5PL, UK; 6 Centre for Cognitive Ageing & Cognitive Epidemiology, Department of Psychology, University of Edinburgh, Edinburgh EH8 9AZ, UK

**Keywords:** vegetarian, pregnancy, cognition, executive function, IQ, child, polyunsaturated fatty acids, cobalamin, docosahexaenoic acid, dietary choice

## Abstract

Compared with omnivorous mothers, vegetarian mothers have lower intakes of some nutrients required for neurological development. However, there is a lack of information about the impact of vegetarianism during pregnancy on subsequent cognitive function in children. The aim of this study was to investigate whether vegetarianism during pregnancy is associated with altered maternal nutritional status and with cognitive function in children at six to seven years of age. Women aged 20–34 years participating in a prospective observational study who provided dietary data and blood samples in early pregnancy (11 weeks; 78 vegetarians and 2144 omnivores) or late pregnancy (34 weeks; 91 vegetarians and 2552 omnivores). Compared with omnivorous women, vegetarian women had lower blood concentrations of arachidonic acid, docosahexaenoic acid, and cobalamin in early and late pregnancy. Vegetarianism in pregnancy was linked to higher maternal educational attainment, longer breastfeeding duration, lower incidence of smoking during pregnancy and a tendency towards higher IQ in the mothers. Concentrations of some nutrients required for neurodevelopment were lower in maternal blood during gestation; however, after controlling for confounders consuming a vegetarian diet during pregnancy was not associated with poorer neurocognitive development of the children in this study.

## 1. Introduction

Optimal development of the human brain requires an adequate supply of nutrients during gestation and childhood [1]. The brain grows particularly rapidly in utero and during the first two years of life, suggesting a period of development which may be vulnerable to inadequate nutrition [2]. Several nutrients have been identified as being particularly important for neurodevelopment and function including iron, cobalamin, iodine and polyunsaturated fatty acids (PUFA). Iron is required for myelination [3] and for the synthesis of neurotransmitters [4]. Iron deficiency can induce hypomyelination of neurones [5,6] and impaired activity of the dopamine-opiate system [4]. The relatively few studies available have shown that iron deficiency in childhood has been associated with impaired cognitive function [7,8,9,10,11]. Cobalamin deficiency in infancy has been associated with hypomyelination [12,13] and combined with folate deficiency, can impair synthesis of cell membranes and neurotransmitters [14] and alter epigenetic process involved in memory formation [15]. Cobalamin deficiency during pregnancy has been associated with lower cognitive scores at two years of age, which was exacerbated by the presence of vitamin B6 deficiency [16]. Triiodothyronine and thyroxine are essential for brain development and the negative effects of severe pre- and post- natal iodine deficiency are well established [17]. There is also emerging evidence that marginal maternal iodine status may adversely affect fetal brain development [18] and we have reported that higher preconception maternal iodine status is positively associated with higher IQ at age 6 to 7 years [19]. The PUFA arachidonic acid (ARA, 20:4n-6) and docosahexaenoic acid (DHA, 22:6n-3) accumulate in phospholipids of the human brain during the third trimester of pregnancy and during the first 2 years of life [20,21]. Diets deficient in omega-3 PUFA fed to pregnant monkeys have been associated with impaired cognitive function and altered behaviour [22,23]. However, the importance of prenatal assimilation of ARA and DHA on cognitive function of children born at term is uncertain. Umbilical cord blood ARA and DHA status at birth were not associated with cognitive development at age 4 years [24] or 7 years [25]; others reported previously that maternal ARA and DHA status in early or late pregnanacy were not associated with IQ or executive function in children at 4 years or 6–7 years [26].

Vegetarian diets are characterised by lower amounts or absence of certain nutrients that are important for fetal brain development [27]. For example, a systematic review of cobalamin status amongst vegetarians found that the prevalence of cobalamin deficiency during pregnancy was 33%, 17% and 39% [28] in the first, second and third trimesters, respectively. 15% of Dutch infants born to vegetarian mothers were found to be deficient in cobalamin [29]. A meta-analysis of 27 studies found that serum ferritin concentration was lower in vegetarian men and non-pregnant women then omnivorous men and women [30]. However, pregnant vegetarian women may be more likely to take iron supplements during the first two trimesters and less likely to have low dietary iron intake than omnivorous women [31]. The impact of maternal vegetarianism on the iron status of infants varies considerably between studies, possibly due, in part, to inclusion of children across wide age ranges [32].

DHA status in vegetarian women has been shown to be 20% to 40% lower compared with omnivorous women [33] and some studies have reported lower DHA status in breast milk from vegans compared with omnivorous women [34,35]. However, the effect of vegetarian or vegan diets on ARA status is less clear [36,37,38]. ARA concentration was similar in erythrocytes from 4-week-old infants of vegan or omnivorous mothers, but DHA concentration was 70% lower in erythrocytes from infants of vegan mothers than those of omnivores [34]. Lakin et al. [39] reported that the proportion of ARA was 16% lower and DHA 5% lower in erythrocytes from vegetarian women who had recently given birth compared with omnivores. Moreover, compared with omnivorous women the proportion in umbilical cord tissue of DHA from vegetarian pregnancies was 36% lower, but ARA did not differ [39].

Thus infants and children of mothers who follow a vegetarian or vegan diet during pregnancy may be at increased risk of impaired neurodevelopment due to restricted intake of nutrients required for brain development compared with those born to omnivorous women. However, to our knowledge there have been no studies of the effect of maternal vegetarian diet during pregnancy on cognitive function in their children.

To address this, we analysed data from women who participated in the Southampton Women’s Survey [40]. Women were classified as either vegetarian or omnivorous, and had their micronutrient and fatty acid status measured in early and late pregnancy to examine the relationship between maternal diet during periods of development of specific brain structures and later cognitive function in their children. Because a vegetarian diet may result in lower intakes of more than one nutrient, we investigated the relationship between maternal vegetarian dietary choice during pregnancy, rather than testing for associations with individual nutrients, and markers of cognitive function in the children at age 6–7 years.

## 2. Materials and Methods

### 2.1. Ethical Statement

The Southampton Women’s Survey (SWS) received ethical approval from the Southampton and South West Hampshire Local Research Ethics Committee (276/97, 307/97, 153/99w, and 10/H0504/30), and all participants gave written informed consent.

### 2.2. Study Sample

The SWS is a prospective cohort study of women aged 20 to 34 years living in the city of Southampton, UK aimed at investigating the effect of the pre- and post- natal influences on the subsequent health of their children [40]. Non-pregnant women were recruited through primary healthcare practices between 1998 and 2002. Those who subsequently became pregnant with singleton fetuses were followed throughout pregnancy; detailed interviews were conducted at 11 and 34 weeks gestation, when fasting blood samples were collected for analysis of nutrient status. Cognitive function of the children was assessed at 6–7 years.

A total of 3158 women became pregnant and delivered a live-born singleton infant within the study period, of whom 2222 provided dietary data in early pregnancy and 2643 provided dietary data in late pregnancy; 2813 women provided dietary data for at least one of these time points. Detailed interviews were conducted in early (median 11.7 (IQR 11.4, 12.2) weeks) and late (34.5 (34.2, 34.8) weeks) pregnancy. Food intake over the preceding three months was assessed using a validated interviewer-administered food frequency questionnaire (FFQ) [41]; prompt cards were used to ensure standardised responses to the FFQ. Vegetarianism was defined as not reporting in the FFQ consuming any meat or fish in the three months preceding the collection of a pregnancy blood sample. This definition is in line with that for ovo-lacto vegetarians described by Davey et al. [42].

At the pre-pregnancy interview, details of mothers’ educational attainment (defined in six groups according to highest academic qualification) were obtained and height was measured with a portable stadiometer (Harpenden; CMS Weighing Equipment Ltd., London, UK) to the nearest 0.1 cm with the head in the Frankfort plane. Weight was measured using calibrated electronic scales (Seca, Hamburg, Germany) to the nearest 0.1 kg (after removal of shoes and heavy clothing or jewellery). These measurements were used to calculate pre-pregnancy body mass index (BMI). Among women who became pregnant, smoking status was ascertained.

### 2.3. Maternal Blood Sample Collection and Analysis of Nutrient Status

Venous blood samples were collected into tubes without anticoagulant in early and late pregnancy. Serum was isolated by centrifugation and stored at −80 °C. Serum phosphatidylcholine (PC) fatty acid composition was measured essentially as described [43]. Briefly, dipentadecanoyl PC (100 µg) internal standard was added to thawed serum (0.8 mL) and total lipids were then extracted with chloroform and methanol [44]. Lipid extracts were dried under N_2_, then dissolved in chloroform (1.0 mL) and applied to a BondElut aminopropylsilica cartridge (100 mg) (Agilent Technologies) [45]. Unbound lipids were removed by washing with chloroform and PC was then eluted with chloroform/methanol (60:40, *v*/*v*). Purified PC was dissolved in toluene and fatty acid methyl esters (FAME) were synthesised by heating at 50 °C in the presence of methanol containing 2% (*v*/*v*) sulphuric acid. FAME were recovered with hexane and resolved on a BPX-70 fused silica capillary column (30 m × 0·22 mm × 25 μm; SGE Analytical Science) using an Agilent 6890 gas chromatograph equipped with flame ionisation detection (Agilent Technologies Ltd., Stockport, Cheshire, UK) [43]. The concentrations of individual fatty acids were calculated from the ratio of their peak areas to the peak area of the internal standard, multiplied by the amount of standard and corrected for the volume of serum extracted.

Micronutrient concentrations during pregnancy were measured in serum stored at −80 °C until analysis. Serum folate and cobalamin concentrations were measured by an automated immunoassay using the Access Immunoassay System on a Beckman Coulter DXi 800 according to the manufacturer’s instructions. Red cell folate concentration was measured by microparticle enzyme immunoassay using an Abbott IMX machine (Abbott, Columbus, OH, USA). Analysis of nicotinamide and riboflavin concentrations was carried out by BEVITAL AS (Bergen, Norway) using liquid chromatography-mass spectrometry. Serum ferritin was measured using an AssayMax Human Ferritin ELISA (AssayPro, St. Charles, MO, USA) according to the manufacturer’s instructions. Haemoglobin concentration was measured as part of a full blood count (Abbott Cell-Dyne). β-carotene concentration was measured by HPLC as described [46]. Briefly, plasma was deproteinised with methanol containing the internal standard (tocopherol acetate), then extracted with heptane. The evaporated heptane layer was reconstituted with mobile phase; methanol, acetonitrile, chloroform (68/20/12 by volume) and injected onto a 50 × 3.0 mm 2 μm column of YMC-Ultra-HT Hydrosphere C18 (YMC, Kyoto, Japan) run at 0.52 mL/min. β-carotene was well resolved during a 6 min run and monitored at 452 nm by a diode array detector. Analytes were calibrated with HPLC grade standards (CaroteNature, Lupsingen, Switzerland). Serum homocysteine concentration was determined by liquid chromatography-tandem mass spectrometry as described [47].

### 2.4. Assessment of Cognitive Function in Children

Cognitive function in the children was assessed as described previously [26]. The Wechsler Abbreviated Scale of Intelligence (WASI) was used to test IQ [48] and the Cambridge Neuropsychological Test Automated Battery (CANTAB^®^, Cambridge Cognition, Cambridge, UK) was used to assess executive function at 6–7 year of age [49]. The assessment of executive function involved four specific tests and focussed on specific outcomes as in our previous work [26]: (1) Visual working memory was tested by delayed matching to sample (DMS), (i) total correct; (2) Rule learning and cognitive flexibility was assessed by intra/extra-dimensional shift (IED), (ii) total errors, (iii) adjusted errors, and (iv) stages completed, (v) pre-extradimensional shift errors, (vi) extradimensional shift errors and (vii) adjusted IED total errors; (3) Working memory was assessed by Spatial Span (SSP) length. (4) Impulsivity and decision making were tested by Information Sampling Task (IST) [50].

### 2.5. Statistical Analysis

Summary statistics are presented as mean (SD) for normally distributed continuous variables or median (IQR) for non-normally distributed continuous variables and percentages for categorical variables. Student’s t-tests (for normally distributed continuous variables), Mann-Whitney U-Tests (for non-normally distributed continuous variables) and chi-squared tests (for categorical variables) were used to compare the distributions of characteristics between omnivorous and vegetarian women. A *p*-value of < 0.05 was considered statistically significant.

IED pre- extra-dimensional shift (EDS) errors, IED EDS errors and IED total errors (adjusted) were all transformed using Fisher-Yates transformations [51], so the resulting variable has SD units. It was not possible to transform IED total errors (stage 1), IED total errors (stage 8) and IST mean probability correct, so these were classified into five groups. Similarly, IED stages completed were in four groups (five groups were inappropriate here due to the distribution of responses). It was not necessary to transform DMS total correct, or SSP span length, which are shown in the original units.

**Figure 1:**
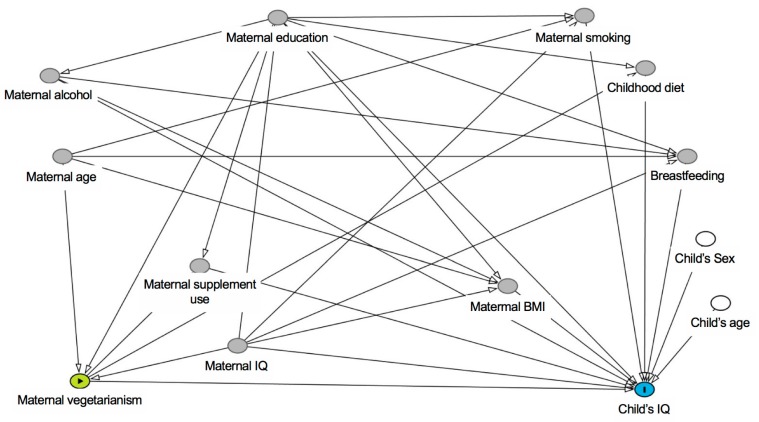
Directed Acyclic Graph (DAG) for the association between maternal vegetarianism and childhood cognitive development. Confounders identified by the DAG were maternal IQ, maternal education and maternal age.

Linear regression models were fitted to assess the association between maternal vegetarianism and childhood cognitive development outcomes. Models were fitted unadjusted and adjusted for confounders. We used the Directed Acyclic Graph (DAG) approach [52] to select suitable confounders (Figure 1). The confounders identified by the DAG for the association between maternal vegetarianism and childhood cognitive development were maternal IQ, maternal education and maternal age. In addition, all models were adjusted for the sex of the child, and CANTAB^®^ outcomes were also adjusted for the child’s age (WASI outcomes are inherently adjusted for age) as competing exposures.

## 3. Results

### 3.1. Participant Characteristics

78 out of 2222 women (3.5%) were identified as vegetarian in early pregnancy and 91 out of 2643 women (3.4%) were identified as vegetarian in late pregnancy. Of the 78 early pregnancy vegetarians, 67 continued this dietary choice into late pregnancy, 4 were not vegetarian in late pregnancy and 7 did not have dietary data at 34 weeks gestation. 96% of the women were taking dietary supplements in early pregnancy, whereas only 49% were taking supplements in late pregnancy. Vegetarian mothers were on average 0.8 years older at the time of their children’s birth than omnivorous mothers in the late pregnancy group, but there was no difference in the age of the mothers in the early pregnancy group (Table 1). There were no differences between omnivorous and vegetarian women in the early or late pregnancy groups in their body-mass-index (BMI) or parity. More omnivorous than vegetarian women smoked during pregnancy, while more vegetarian women had passed advanced school examinations (A levels) compared with omnivorous women (Table 1). Amongst mothers who had their IQ measured when their children were aged 6–7, IQ was marginally higher amongst those who chose a vegetarian diet than those who followed a omnivorous diet. Vegetarian women breast-fed their infants for an average of 13 to 14 weeks longer than omnivorous women. In early pregnancy there was no difference between the proportion of mothers who took supplements; in late pregnancy a higher proportion of vegetarian (69.2%) than omnivorous (48.0%) mothers took supplements.

Gestational age at birth was slightly greater among those born to vegetarians than to those of omnivores (40.8 versus 40.1 weeks, Table 1) but there were no differences in child’s BMI between children born to omnivorous or vegetarian mothers (Table 1).

### 3.2. Serum Phosphatidylcholine Fatty Acid Concentrations in Pregnant Women

Twenty-two fatty acids were measured routinely in serum PC (Table 2). Women who were vegetarians in early pregnancy had lower concentrations than omnivorous women for all fatty acids except 22:0, 22:4n-6, 22:5n-6 and 24:1n-9. In particular, eicosapentaenoic acid (20:5n-3; EPA), and DHA concentrations were considerably lower in vegetarians than in omnivorous women.

The differences in serum PC fatty acid composition between women who were vegetarians in late pregnancy and those who were omnivorous were similar to those in early pregnancy (Table 2), but the differences for 22:5n-3, 22:5n-6, 20:1n-9 and 18:1n-7 were stronger than seen in early pregnancy (Table 2).

### 3.3. The Concentrations of Micronutrients in Serum from Vegetarian and Omnivorous Women

Serum cobalamin concentration was lower in vegetarian women in both early and late pregnancy compared with omnivorous women (Table 3), but there was no difference in serum homocysteine concentration between the dietary groups at either time point in pregnancy. At both time points the haemoglobin levels of vegetarians was 3.0 g/L lower than in omnivores. In early pregnancy, erythrocyte folate concentration was somewhat higher in vegetarian women than in omnivorous women, but this measurement was not available from the late pregnancy samples, in which serum folate was assessed; this was found to be higher in the vegetarian women.

### 3.4. The Relationship between Maternal Dietary Choice and Cognitive Function in Their Children

The results of linear regression models with vegetarian dietary choice during pregnancy as the predictor and measures of the children’s IQ and cognitive function as the outcome (unadjusted and adjusted) are summarised in Table 4. Maternal vegetarianism in either early or late pregnancy was not associated with reductions in IQ at ages 6 to 7 years (*β* = 3.41 (95% CI −3.46, 10.29), *p* = 0.33, *β* = 1.34 (95% CI −4.75, 7.43), *p* = 0.66, respectively). Maternal vegetarianism was not associated with detrimental effects on CANTAB cognitive outcomes, but some associations of borderline statistical significance were seen with enhanced CANTAB outcomes; for example CANTAB IED errors (adjusted) were lower in offspring of vegetarian mothers in early pregnancy (*β* = −0.48 (95% CI −0.94, −0.01), *p* = 0.04) and late pregnancy (*β* = −0.36 (95% CI −0.79, 0.06), *p* = 0.09).

## 4. Discussion

Consuming a diet that excludes meat and fish may result in lower intakes of nutrients that are important for brain development and function; the impact of such dietary choice during pregnancy on cognitive function in the children has not been investigated previously. As expected, the present study showed that consuming a vegetarian diet during pregnancy was associated with lower maternal concentrations of cobalamin and some PUFA that are required for normal brain development and function. Nevertheless, the findings show that a vegetarian diet during pregnancy had no discernible negative effects on the cognitive function of the children at 6 to 7 years of age.

Approximately 3% of women who participated in the study were classified as vegetarian. As others have shown [42,54], the vegetarian women tended to have higher IQ and educational attainment, breastfed for longer and had a lower incidence of tobacco smoking during pregnancy than the omnivorous women. As reported previously [28,55], cobalamin concentration was lower in vegetarian women irrespective of the timing of sample collection. In contrast, serum folate concentration was higher in vegetarian women compared with omnivorous women. This is consistent with the reported higher folate intakes in pregnant vegetarians [56]. Although higher maternal folate may be considered beneficial for neurodevelopment, imbalance between cobalamin and folate status may impair 1-carbon metabolism [57] which, in turn, could impair epigenetic processes in the central nervous including those involved in memory [58]. However, since there was no difference in serum homocysteine concentration between vegetarian and omnivorous women in early or late pregnancy, any effect of the differences in relative folate and cobalamin concentrations between dietary groups appears to be modest.

Previous reports of fatty acid status in vegetarians during pregnancy have mainly restricted their findings to omega-3 PUFA. For example, combined EPA plus DHA intake was 94% lower in vegetarian women who had recently given birth compared with omnivorous women, while the proportions of EPA and DHA were lower in placental lipids from vegetarian compared to omnivorous pregnancies [38]. Importantly, the proportion of DHA in breast milk from vegetarian women was 62% lower than in milk from omnivorous women, while DHA concentration in umbilical cord blood serum from UK Hindu vegetarians was 32% lower than in omnivorous pregnancies [35]. Thus maternal DHA supply to the child appears to be reduced in vegetarian pregnancies both before birth and in infancy which may represent increased risk of impaired neurodevelopment [21]. The present findings agree with previous reports by showing lower EPA and DHA concentrations in blood from vegetarian compared with omnivorous women. However, this study extends these observations by providing a comprehensive analysis of serum PC fatty acids in a substantially greater number of pregnancies than have been reported previously [34,35,39]. The main differences in serum PC fatty acids between vegetarian and omnivorous women were in PUFA concentrations. In addition to EPA and DHA, the concentrations of 20:4n-3 and of 20:4n-6, which is also an important structural component of neural membranes [20] and is obtained from meat, were lower in vegetarian than omnivorous women. In contrast, 22:5n-6 concentration was higher in serum PC from vegetarian than from omnivorous women. 22:5n-6 is a desaturation and elongation product of 20:4n-6 [59] that is enriched in tissues during low omega-3 PUFA intakes and is associated with impaired neural function [59]. This suggests that the lower EPA and DHA status in vegetarian women was sufficient to induce changes to PUFA metabolism that have been associated with omega-3 PUFA deficiency. It is possible that conversion to ARA to 22:5n-6 may have contributed to lower ARA status.

Vegetarian dietary choice was associated with differences compared with omnivorous women in the concentrations of cobalamin and fatty acids in maternal blood that are important for brain development and function. Since neurological development and function may be influenced by the net availability of such nutrients, we captured the effect of maternal vegetarian diet on the children’s cognitive function in terms of maternal dietary choice rather than individual nutrients. The period of rapid brain growth in early life is associated with temporal differences in the development of individual brain structures [2]. It was, therefore, possible that any association between maternal dietary choice and cognitive function in the children could be related to the time of sampling.

Despite lower maternal concentrations of nutrients that are involved in neurological development, in particular cobalamin, DHA and ARA, there were no negative associations between maternal dietary choice in early or late pregnancy and WASI IQ or measures of executive function in the children at 6–7 years after adjustment for confounders including maternal education and IQ. One possible explanation is that some unmeasured nutritional or non-nutritional factors in vegetarians may have had counterbalancing positive effects on cognitive development. Although the DAG approach captured major potential confounders, such as maternal education and IQ, there may be residual confounding. If, for example, maternal education does not completely capture socioeconomic status, then enhanced socioeconomic conditions associated with vegetarian lifestyles could compensate for any negative effects of lower nutrient status on childhood cognitive function. Residual confounding could also explain the association of maternal vegetarianism with somewhat enhanced CANTAB outcomes. One additional consideration is that although concentrations of specific nutrients were lower in vegetarian than omnivorous women, these were still within ranges associated with uncomplicated pregnancies [60]. Hence, the magnitude of the deficit may not have been sufficient to induce measurable effects on childhood cognition.

The main limitations of the study are that the data presented here were collected as part of a larger ongoing project investigating the impact of the environment in early life of subsequent health [40], which limits the strength of the conclusions that can be drawn because of the design was not optimised to address the aims of the present study. The number of vegetarian women who took part was markedly lower than the number of omnivorous women and the study may not have had sufficient statistical power to detect a clinically important effect size. Nonetheless, to our knowledge this is the largest study to date that has investigated the effect of consuming a vegetarian diet during pregnancy on direct measures of cognitive function in children. Some metabolites were not measured in both early and late pregnancy. Importantly, our analyses did not investigate the impact of postnatal environmental factors, including diet, on the children’s cognitive function.

## 5. Conclusions

In conclusion, the findings of this study suggest that consuming a vegetarian diet during pregnancy does not adversely affect the neurocognitive development of the children, provided maternal concentrations of nutrients that are required for neurological development are within usual ranges.

## Figures and Tables

**Table 1:** Characteristics of mothers and children by maternal dietary choice.

	Vegetarian		Omnivorous		*p*
	n	Statistic	n	Statistic	
Mother	**Early pregnancy**				
Age at child’s birth (y), mean (SD)	78	30.4 (3.4)	2142	30.5 (3.7)	0.85
IQ (measured when child 6–7 years old), mean (SD)	18	113.7 (12.1)	530	107.5 (12.7)	0.04
Educational attainment; qualifications ≥ A-level, n (%)	78	61 (78.2)	2138	1266 (59.2)	0.001
Smoked in pregnancy, n (%)	78	5 (6.4)	2114	335 (15.8)	0.02
BMI (kg/m^2^), median (IQR)	78	23.9 (22.1, 25.4)	2128	24.3 (21.9, 27.5)	0.24
Multiparous, n (%)	78	35 (44.9)	2142	1047 (48.9)	0.49
Duration breastfeeding (weeks), median (IQR)	71	29.9 (23.5)	1987	17.1 (21.1)	<0.001
Supplement use, n (%)	78	76 (97.4)	2143	2047 (95.5)	0.58
Child					
Female, n (%)	16	6 (37.5)	479	237 (49.5)	0.35
Gestational age at birth (weeks), median (IQR)	16	40.8 (40.1, 41.4)	479	40.1 (39.0, 41.1)	0.04
BMI at 6–7 y (kg/m^2^), median (IQR)	16	15.9 (14.7, 16.6)	470	15.8 (14.9, 16.8)	0.88
Age at 6–7 y follow-up (y), mean (SD)	16	7.1 (0.3)	479	7.0 (0.2)	0.12
Mother	**Late pregnancy**				
Age at child’s birth (y), mean (SD)	91	31.3 (3.4)	2551	30.5 (3.8)	0.04
IQ (measured when child 6–7 years old), mean (SD)	23	112.0 (11.6)	583	107.4 (12.9)	0.10
Educational attainment; qualifications ≥ A-level, n (%)	91	71 (78.0)	2546	1464 (57.5)	<0.001
Smoked in pregnancy, n (%)	91	6 (6.6)	2544	443 (17.4)	0.007
BMI (kg/m^2^), median (IQR)	91	23.9 (22.1, 25.7)	2531	24.2 (21.9, 27.5)	0.50
Multiparous, n (%)	91	48 (52.7)	2551	1326 (52.0)	0.89
Duration breastfeeding (weeks), median (IQR)	85	31.3 (25.8)	2373	17.3 (21.6)	<0.001
Supplement use	91	63 (69.2)	2552	1226 (48.0)	<0.001
Child					
Female, n (%)	22	6 (27.3)	524	255 (48.7)	0.05
Gestational age at birth (weeks), median (IQR)	22	40.6 (40.0, 41.2)	524	40.1 (39.2, 41.2)	0.08
BMI at 6–7 y (kg/m^2^), median (IQR)	22	15.3 (14.7, 15.9)	510	15.8 (14.9, 16.9)	0.24
Age at 6–7 y follow-up (y), mean (SD)	22	7.1 (0.2)	524	7.0 (0.2)	0.10

Sample sizes varied for specific variables because of item-specific missing values. *p* values were determined using Student’s *t*-test for normally distributed variables, the Mann-Whitney U Test for non-normally distributed variables and chi-squared tests for categorical variables (or Fisher’s exact test for early pregnancy supplement use due to small numbers).

**Table 2:** Serum fatty acid concentrations in early and late pregnancy vegetarians and omnivores.

	Fatty Acid Concentration (μg/mL)					
	Early Pregnancy			Late Pregnancy		
	Vegetarians (n = 32)	Omnivores (n = 967)	*p*	Vegetarians (n = 59)	Omnivores (n = 1703)	*p*
14:0	4.8 (3.7, 7.5)	6.0 (4.6, 7.8)	0.07	3.2 (2.0, 4.9)	3.2 (2.2, 4.5)	0.40
16:0	489.5 (420.9, 594.0)	536.8 (458.9, 639.0)	0.06	462.5 (381.5, 569.4)	514.8 (399.6, 631.8)	0.04
16:1n-7	7.1 (5.6, 11.6)	9.3 (6.9, 12.8)	0.05	5.8 (3.3, 8.9)	5.2 (3.3, 8.4)	0.63
18:0	185.5 (161.1, 219.9)	204.6 (171.9, 238.2)	0.02	136.4 (97.5, 169.2)	143.9 (111.2, 178.8)	0.07
18:1n-9	158.9 (132.6, 194.1)	177.9 (148.9, 212.6)	0.08	148.9 (110.4, 192.1)	170.2 (130.3, 210.3)	0.01
18:1n-7	28.8 (24.5, 31.4)	29.9 (24.2, 35.8)	0.19	17.7 (12.7, 21.9)	20.3 (13.9, 26.0)	0.007
18:2n-6	367.2 (317.2, 428.9)	368.5 (312.0, 435.5)	0.97	328.3 (248.2, 387.0)	333.6 (258.7, 408.3)	0.60
18:3n-6	1.4 (1.0, 2.4)	1.7 (1.3, 2.4)	0.07	0.0 (0.0, 0.0) †	0.0 (0.0, 0.0) †	0.19
18:3n-3	5.9 (4.0, 8.9)	6.1 (4.5, 8.1)	0.53	3.7 (2.7, 5.2)	4.0 (3.0, 5.6)	0.21
20:0	3.9 (2.5, 5.6)	4.2 (3.1, 5.6)	0.30	2.1 (0.0, 3.1)	2.3 (0.0, 3.2)	0.36
20:1n-9	3.7 (3.1, 5.0)	3.8 (3.0, 4.8)	0.91	1.4 (0.0, 2.3)	2.0 (0.0, 2.7)	0.007
20:2n-6	7.6 (5.3, 9.3)	8.0 (6.6, 9.8)	0.30	4.9 (3.7, 6.1)	5.1 (3.9, 6.5)	0.26
20:3n-6	58.2 (49.7, 79.2)	67.7 (53.7, 83.9)	0.13	52.4 (38.2, 64.8)	53.6 (39.6, 68.6)	0.26
20:4n-6	143.9 (134.8, 170.1)	165.8 (135.5, 200.8)	0.02	100.9 (72.9, 122.7)	108.4 (83.8, 139.0)	0.03
22:0	2.1 (0.9, 2.9)	1.8 (1.1, 2.7)	0.60	0.0 (0.0, 0.0) †	0.0 (0.0, 0.0) †	0.47
20:4n-3	3.1 (2.3, 5.5)	4.2 (2.9, 5.9)	0.04	0.0 (0.0, 2.3)	1.8 (0.0, 2.9)	0.02
20:5n-3	8.4 (6.1, 10.4)	14.0 (10.4, 18.8)	<0.001	3.5 (2.0, 4.6)	5.3 (3.7, 7.8)	<0.001
22:4n-6	7.4 (5.0, 9.2)	6.9 (5.2, 8.6)	0.51	3.4 (2.4, 4.4)	3.3 (2.4, 4.3)	0.74
22:5n-6	7.1 (6.0, 10.4)	6.2 (4.5, 8.3)	0.01	5.2 (3.9, 7.3)	4.3 (3.2, 6.0)	0.001
24:1n-9	4.2 (1.8, 5.3)	3.2 (2.1, 5.1)	0.63	0.0 (0.0, 0.0) †	0.0 (0.0, 0.0) †	0.18
22:5n-3	13.3 (10.6, 16.9)	15.2 (12.3, 19.6)	0.01	5.1 (3.6, 6.5)	6.6 (4.8, 8.8)	<0.001
22:6n-3	57.8 (50.2, 62.9)	82.6 (66.4, 102.7)	<0.001	38.0 (27.9, 48.9)	54.3 (41.2, 72.3)	<0.001

Values are median (IQR); *p* values were determined using the Mann-Whitney U Test. † Undetectable in more than 75% of samples.

**Table 3:** Concentrations of specific micronutrients in early and late pregnancy vegetarian and omnivorous women.

	Early Pregnancy					Late Pregnancy				
	n	Vegetarians	n	Omnivores	*p*	n	Vegetarians	n	Omnivores	*p*
β-carotene (μmol/L)	29	0.4 (0.3, 0.7)	876	0.4 (0.3, 0.6)	0.52	48	0.3 (0.3, 0.5)	1299	0.3 (0.2, 0.5)	0.15
Cobalamin (pg/mL)	71	292.6 (211.3, 358.8)	1910	368.7 (279.6, 464.1)	<0.001	78	131.5 (99.0, 182.0)	1982	162.0 (128.0, 209.0)	<0.001
Serum folate (μg/L)	71	ND		ND		78	13.2 (8.9, 21.8)	1940	9.9 (6.5, 17.4)	0.001
Erythrocyte folate (nmol/L)	61	1190 (891, 1400)	1624	1061 (802, 1381)	0.06		ND		ND	
Homocysteine (μmol/L)	71	13.3 (8.7, 19.8)	1904	12.4 (7.8, 20.3)	0.50	14	5.9 (5.3, 7.4)	481	6.1 (5.2, 7.4)	0.63
Riboflavin (nmol/L)		ND		ND		14	14.9 (10.8, 27.6)	481	17.1 (11.9, 25.3)	0.74
Nicotinamide (nmol/L)		ND		ND		14	118.9 (36.6, 209.9)	481	140.2 (82.6, 213.6)	0.24
Haemoglobin (g/L)	69	124.0 (119.0, 131.0)	1851	127.0 (121.0, 133.0)	0.09	87	112.0 (105.0, 119.0)	2192	115.0 (109.0, 122.0)	<0.001
Ferritin (ng/mL)		ND		ND		15	46.8 (28.3, 78.1)	484	48.7 (32.6, 75.4)	0.58

Values are median (IQR); *p* values were determined using Mann-Whitney U Tests. ND, Not determined. Reference concentrations of cobalamin and serum folate derived from women with apparently uncomplicated pregnancies: cobalamin, first trimester 118–438 (pg/mL); third trimester 99–526 (pg/mL); serum folate, first trimester 2.6–15.0 ng/mL; third trimester 1.4–20.7 ng/mL [53].

**Table 4:** Maternal dietary choice as predictor of cognitive outcomes in children at 6 to 7 years.

	Unadjusted			Adjusted#		
	n	*β* (95% CI)	*p*	n	*β* (95% CI)	*p*
IQ (WASI)						
Early pregnancy vegetarian, Y/N	564	7.07 (−0.42, 14.55)	0.06	537	3.41 (−3.46, 10.29)	0.33
Late pregnancy vegetarian, Y/N	619	4.01 (−2.60, 10.62)	0.23	594	1.34 (−4.75, 7.43)	0.66
CANTAB^®^ DMS total correct (12 sec delay)						
Early pregnancy vegetarian, Y/N	494	0.53 (−0.07, 1.12)	0.08	479	0.54 (−0.07, 1.14)	0.08
Late pregnancy vegetarian, Y/N	546	0.38 (−0.13, 0.88)	0.15	532	0.41 (−0.11, 0.93)	0.12
CANTAB^®^ IED pre-ED errors (z-score)						
Early pregnancy vegetarian, Y/N	495	0.08 (−0.39, 0.56)	0.73	480	0.11 (−0.37, 0.59)	0.66
Late pregnancy vegetarian, Y/N	545	−0.05 (−0.46, 0.36)	0.80	531	−0.06 (−0.47, 0.36)	0.79
CANTAB^®^ IED EDS errors						
Early pregnancy vegetarian, Y/N	495	−0.51 (−1.00, −0.03)	0.04	480	−0.48 (−0.96, −0.00)	0.05
Late pregnancy vegetarian, Y/N	545	−0.39 (−0.81, 0.02)	0.07	531	−0.33 (−0.75, 0.09)	0.12
CANTAB^®^ IED total errors (stage 1) in 5 groups						
Early pregnancy vegetarian, Y/N	492	−0.39 (−0.95, 0.18)	0.18	477	−0.37 (−0.94, 0.19)	0.20
Late pregnancy vegetarian, Y/N	540	−0.21 (−0.70, 0.28)	0.41	526	−0.15 (−0.64, 0.35)	0.56
CANTAB^®^ IED total errors (stage 8) in 5 groups						
Early pregnancy vegetarian, Y/N	492	−0.73 (−1.43, −0.03)	0.04	477	−0.65 (−1.34, 0.04)	0.07
Late pregnancy vegetarian, Y/N	540	−0.61 (−1.20, −0.01)	0.05	526	−0.49 (−1.08, 0.11)	0.11
CANTAB^®^ IED total errors (adjusted)						
Early pregnancy vegetarian, Y/N	495	−0.58 (−1.06, −0.11)	0.02	480	−0.48 (−0.94, −0.01)	0.04
Late pregnancy vegetarian, Y/N	545	−0.46 (−0.88, −0.04)	0.03	531	−0.36 (−0.79, 0.06)	0.09
CANTAB^®^ IED stages completed in 4 groups						
Early pregnancy vegetarian, Y/N	495	0.53 (0.08, 0.97)	0.02	480	0.43 (−0.00, 0.86)	0.05
Late pregnancy vegetarian, Y/N	545	0.46 (0.08, 0.84)	0.02	531	0.34 (−0.04, 0.72)	0.08
CANTAB^®^ SSP span length						
Early pregnancy vegetarian, Y/N	468	−0.05 (−0.50, 0.40)	0.83	453	−0.12 (−0.56, 0.31)	0.58
Late pregnancy vegetarian, Y/N	521	−0.09 (−0.47, 0.28)	0.63	507	−0.19 (−0.56, 0.18)	0.32
CANTAB^®^ IST mean prob. correct (win condition fixed) in 5 groups						
Early pregnancy vegetarian, Y/N	452	0.56 (−0.18, 1.30)	0.14	438	0.52 (−0.22, 1.27)	0.17
Late pregnancy vegetarian, Y/N	486	0.24 (−0.39, 0.86)	0.46	473	0.20 (−0.43, 0.83)	0.53

# Adjusted for maternal age, maternal education, maternal IQ, sex and, for Cambridge Neuro-psychological Test Automated Battery (CANTAB^®^) outcomes, child’s age. DMS, delayed matching to sample; IED, intra/extra dimensional shift; IST, Information Sampling Task; SSP, spatial span length; WASI, Weschler Abbreviated Scale of Intelligence. Values are linear regression coefficients (*β*) and 95% confidence intervals.

## Abbreviations

ARA	Arachidonic acid
BMI	Body-mass-index
CANTAB^®^	Cambridge Neuropsychological Test Automated Battery
DAG	Direct acyclic graph
DHA	Docosahexaenoic acid
DMS	Delayed matching to sample
FAME	Fatty acid methyl esters
FFQ	Food frequency questionnaire
IED	Intra/extra-dimensional shift
IST	Information Sampling Task
PC	Phosphatidylcholine
SSP	Spatial Span
WASI	Wechsler Abbreviated Scale of Intelligence
WPPSI	Wechsler Preschool and Primary Scale of Intelligence.
